# Slower carriers limit charge generation in organic semiconductor light-harvesting systems

**DOI:** 10.1038/ncomms11944

**Published:** 2016-06-21

**Authors:** Martin Stolterfoht, Ardalan Armin, Safa Shoaee, Ivan Kassal, Paul Burn, Paul Meredith

**Affiliations:** 1 Centre for Organic Photonics & Electronics, School of Mathematics and Physics, School of Chemistry and Molecular Biosciences, The University of Queensland, Brisbane, Queensland 4072, Australia; 2 Centre for Engineered Quantum Systems, Centre for Quantum Computation and Communication Technology, School of Mathematics and Physics, The University of Queensland, Brisbane, Queensland 4072, Australia

## Abstract

Blends of electron-donating and -accepting organic semiconductors are widely used as photoactive materials in next-generation solar cells and photodetectors. The yield of free charges in these systems is often determined by the separation of interfacial electron–hole pairs, which is expected to depend on the ability of the faster carrier to escape the Coulomb potential. Here we show, by measuring geminate and non-geminate losses and key transport parameters in a series of bulk-heterojunction solar cells, that the charge-generation yield increases with increasing slower carrier mobility. This is in direct contrast with the well-established Braun model where the dissociation rate is proportional to the mobility sum, and recent models that underscore the importance of fullerene aggregation for coherent electron propagation. The behaviour is attributed to the restriction of opposite charges to different phases, and to an entropic contribution that favours the joint separation of both charge carriers.

## Abstract (web summary)

In organic solar cells, the photogeneration of free charge carriers is limited by the dissociation of interfacial charge transfer states. Here, the authors study the impact of charge carrier mobilities in operational devices and show that the slowest charge carriers limit the dissociation of charge transfer states.

## Graphical abstract

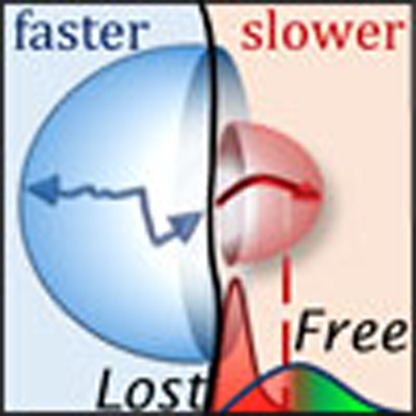

Charge generation in electron donor:acceptor blends of excitonic organic semiconductors is complex and still not fully understood^1^,^2^. The blends form molecular heterojunctions, and the so-called bulk-heterojunction (BHJ) where the organic semiconductors create nanoscale interconnected neat and mixed phases^3^ is the preferred and dominant architecture in organic solar cells and photodiodes. Although a complete understanding of the critical mechanisms and pathways from the photoexcitation to free charges remains elusive, an increasing body of evidence suggests that it is the dissociation of charge transfer (CT) states that defines the charge generation efficiency and overall performance of organic solar cells^4^,^5^,^6^,^7^,^8^. In order to establish strategies to optimize the dissociation of CT states, it is of particular importance to understand how the carrier mobilities affect the separation dynamics.

Braun's model^9^, based on Onsager's theory of ionic dissociation^10^, describes charge generation in donor:acceptor systems as depending on the kinetic competition between the dissociation (*k*_d_) and decay (*k*_f_) rate constants of CT states. The recombination from the charge-separated (CS) state back to the CT state with rate constant *k*_r_ is assumed to be described by the Langevin theory^11^,^12^, which, in a single material phase, predicts that *k*_r_ should be proportional to the sum *μ*_e_+*μ*_h_ of electron and hole mobilities because both carriers drift towards each other due to their electric fields. Another key assumption of the Braun model is that dissociation and recombination are related by detailed balance. Since detailed balance requires that the ratio *k*_d_/*k*_r_ equals the equilibrium constant *K* for charge separation, Braun concluded that *k*_d_ must also be proportional to *μ*_e_+*μ*_h_. If the charge carriers can move away from the interface as quickly as they can return, *k*_d_ and the (geminate) *k*_r_ depend, in the same way, on the carrier mobilities.

The situation is more complicated in a BHJ. Since recombination in a BHJ occurs at the donor:acceptor interface, the faster carrier (*f*) has to wait at the interface for the slower carrier (*s*) before recombination is possible. As a result, Blom and Koster proposed that the arrival of the slower carrier should be the recombination rate-limiting step, giving *k*_r_∝*μ*_s_, the mobility of the slower carriers^13^,^14^,^15^. Following Braun's thesis that dissociation and recombination are opposite processes, this reasoning would suggest that CT state dissociation yield in BHJs should also depend on the slower carrier mobility. However, Blom and Koster's premise does not consider the influence of the domain size on the recombination rate, and this has been investigated recently in ref. 16, wherein the authors showed that the Langevin and Blom–Koster rates are relevant in the limits of very small and very large domains, respectively.

Despite the importance of these kinetic considerations on the charge carrier separation, most studies have focused on the impact of the donor and acceptor energy levels^17^,^18^,^19^ and nanoscale morphology^20^,^21^,^22^,^23^,^24^,^25^. For example, Gélinas *et al*.^24^ have shown that a high fullerene loading is crucial for fullerene aggregation, which assists ultrafast charge separation by enabling electron delocalization. Fullerenes are the dominant *n*-type organic semiconductor and present particularly intriguing challenges in understanding their basic physics because of their molecular size and symmetry. Gélinas *et al*.^24^ concluded—as have others^4^,^26^,^27^—that the faster electrons determine the charge-generation yield, arguing that they can escape via delocalization, leaving the slower hole free to diffuse away at its own pace. This prediction is consistent with Braun's assertion of *k*_d_ determined by *μ*_f_+*μ*_s_, provided that *μ*_f_≫*μ*_s_, as is often the case.

Here we experimentally explore the correlation between the generation yield of free charges and their mobilities, similar to a previous study^28^, and thereby test Braun's model in BHJ solar cells with varying blend compositions. We do so by decoupling geminate and non-geminate recombination using recently introduced experimental methods applied to operational devices^29^,^30^,^31^. Our results show that the dissociation efficiency of CT states is not dependent on *μ*_e_+*μ*_h_, as predicted by Braun's theory, but is instead governed by the slower carriers, whether they are the electrons or the holes. We describe the local dynamics of separating CT states by taking into account the interface that breaks the translational symmetry, changes in the donor:acceptor domain size, and entropic effects that favour the movement of both carriers away from the interface, and not just the escape of the faster carrier.

## Results

### Studied systems

We studied two archetypal BHJ organic solar cells using a polymeric donor with a fullerene acceptor, [(poly[*N*-9′′-heptadecanyl-2,7-carbazole-*alt*-5,5-(4′,7′-di-2-thienyl-2′,1′,3′-benzothiadiazole)]:[6,6]-phenyl-C_70_-butyric acid methyl ester (PCDTBT:PC70BM)^32^ and poly[(4,8-bis{2-ethylhexyloxy}benzo[1,2-b:4,5-b′]dithiophene-2,6-diyl)(3-fluoro-2-{[2–ethylhexyl]carbonyl}thieno[3,4–b]thiophenediyl)] (PTB7):PC70BM^33^. The solar cell fabrication details are provided in the Methods. The results for PCDTBT:PC70BM are shown below. For the PCDTBT:PC70BM blends we varied the composition ratio from 0.1 wt% to 95 wt% PCDTBT in PC70BM, and for the PTB7:PC70BM blends we used 10 wt% to 95 wt% PTB7 in PC70BM. Varying the blend ratio allows one to tune the efficiencies of charge generation and collection in a systematic way. Average current density versus voltage (*JV*) scans were obtained under standard AM 1.5-G illumination and are provided in Supplementary Figs 1 and 2, and Supplementary Tables 1 and 2 for the PCDTBT:PC70BM and PTB7:PC70BM blends, respectively. As the composition ratio varies, the power conversion efficiency is predominantly determined by differences in the short-circuit current density (*J*_SC_).

### Photogeneration yields of all blend compositions

**Figure 1:**
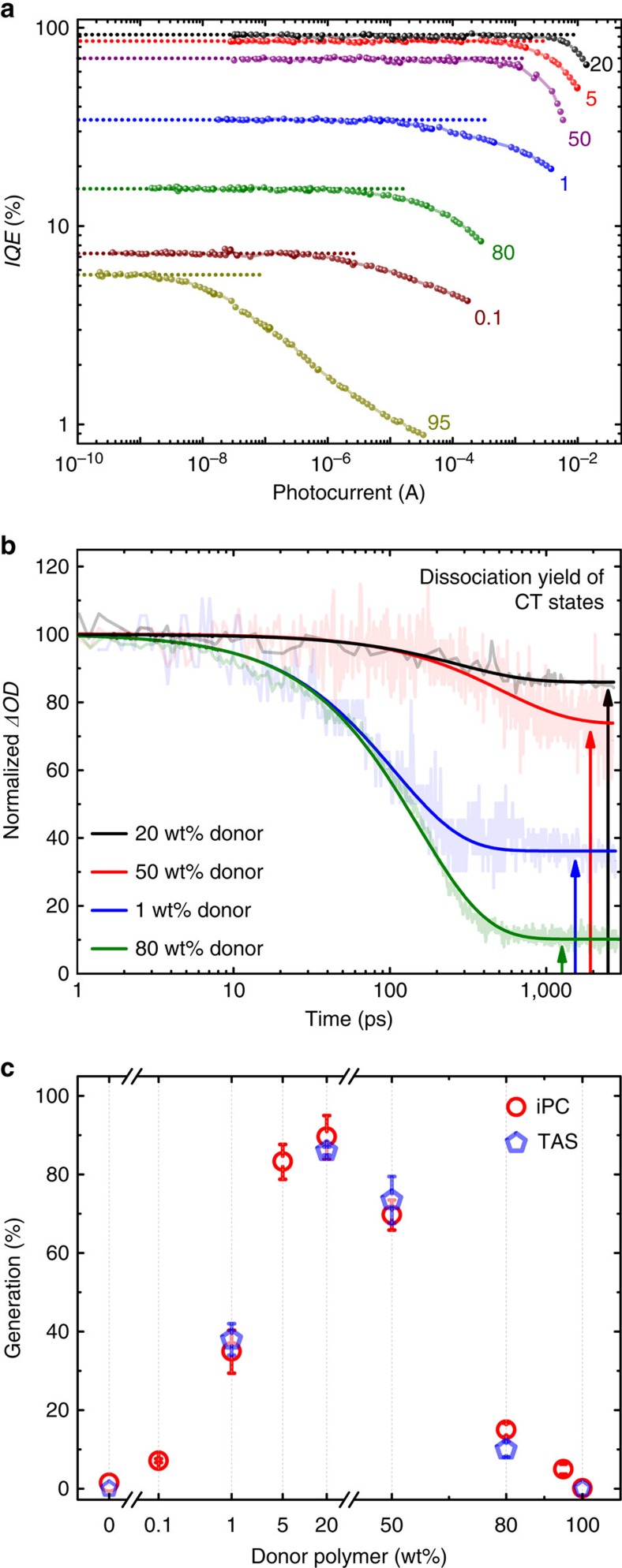
Charge-generation yields from iPC and TAS. (**a**) IQEs as a function of the photocurrent of PCDTBT:PC70BM devices with different donor fractions (wt%, marked by the numbers). The constant IQE at low photocurrents (as marked by the dotted lines) before non-geminate recombination causes the downward deviation, is an estimate of the charge-generation yield. (**b**) Normalized transient absorption spectra of the blends monitoring the polymer positive polarons, following excitation at 560 nm at a fluence of 500 nJ cm^−2^. Solid lines are single exponential fits, indicating a geminate decay of bound and free charges, which absorb at 1,000 nm. The dissociation yield of the CT states is given by the fraction of charges remaining at long times (as marked by the arrows). (**c**) The generation efficiency of free charges determined from **a**,**b** as a function of the donor fraction. iPC error bars are determined from the first s.d. of the constant IQE regime considering two measurements on different pixels and a relative variation of 5% of the active layer absorption. TAS error bars represent the first s.d. of the plateau regime of the transient absorption signals.

To study the relation between charge generation and the carrier mobilities, we performed intensity-dependent internal quantum efficiency (IQE) measurements. In Fig. 1a we plot the IQE as calculated from the intensity-dependent photocurrent (iPC; Supplementary Fig. 3) and the actual photojunction absorptions in operational devices (Supplementary Fig. 4) versus the photocurrent at an excitation wavelength of 532 nm. The results for PTB7:PC70BM are shown in Supplementary Fig. 5. These measurements have allowed us to quantify the combined efficiencies of carrier photogeneration and extraction, and to decouple first- and higher-order photocurrent losses with respect to the incident light intensity. By increasing the light intensity, the photocurrent can reach the slower carrier space-charge-limited photocurrent (*I*_SCLC_), where bimolecular (non-geminate) recombination of oppositely charged carriers starts to strongly influence the charge extraction efficiency^29^. This is seen as an IQE that decreases at higher light intensities (or photocurrents). We note that the recombination of free charges with trapped charges is bimolecular in nature, and is thus distinct in the iPC measurements^30^. Moreover, the equilibrium charge carrier density is low in all studied devices (much less than *CU*_BI_, where *C* is the device capacitance and *U*_BI_ the built-in voltage), which prevents significant pseudo-first-order recombination between free and equilibrium carriers. Therefore, charge extraction free of non-geminate losses in the bulk can be realized if the light intensity is sufficiently low to guarantee a photocurrent lower than the *I*_SCLC_. If geminate recombination losses of free carriers or losses because of back diffusion into the reverse electrode^34^,^35^ are considered to be minimal, then the charge generation can be readily quantified from the constant IQE value^30^.

To corroborate the electrical measurements of the charge-generation efficiency, we employed transient absorption spectroscopy (TAS) to monitor the populations of photogenerated bound and free charge carriers following low-intensity laser excitation, as shown in Fig. 1b. At low intensities, the signals exhibit intensity-independent exponential decay dynamics, indicating first-order losses in the bound-charge states, which is consistent with a previous report^36^. The dissociation yield of the CT states is obtained from the fraction of free charges remaining at long times (see Methods and Supplementary Fig. 6 for further details).

The estimated photocarrier generation yields from iPC are in good agreement with the CT dissociation yields from TAS as shown in Fig. 1c. Furthermore, all the blends studied by TAS showed similar spectra but very short exciton lifetimes compared with neat PCDTBT, suggesting efficient exciton dissociation (see Supplementary Table 3). Therefore, the geminate recombination losses obtained by iPC can be largely attributed to CT state recombination losses^37^, as opposed to exciton losses. In line with previous studies^38^ and our *JV* measurements, we observe that geminate recombination losses are minimized at a polymer loading of 20 wt%.

### Carrier mobilities of all blend compositions

To correlate the generation efficiency with charge carrier mobilities, we determined the mobilities using two independent methods: first, resistance-dependent photovoltage (RPV), which allows to directly monitor the arrival of extracted charges on the device electrodes^31^ (see Methods, Supplementary Figs 7 and 8); and, second, iPC to estimate the charge-extraction-limiting slower carrier mobility. Figure 1a shows that *I*_SCLC_ varies from 10^−8^ to almost 10^−2 ^A in the 95 wt% and the 20 wt% PCDTBT blends, respectively. The *I*_SCLC_ is proportional to the product of the slower carrier mobility and the square root of the reduction factor of the Langevin recombination coefficient (*μ*_s_*γ*^1/2^) (ref. 29). RPV measurements at high laser intensities^39^ reveal Langevin recombination in blends with imbalanced donor:acceptor concentrations (*γ*∼1) and non-Langevin recombination in efficient blends (*γ*∼25 to 33; Supplementary Fig. 9).

**Figure 2:**
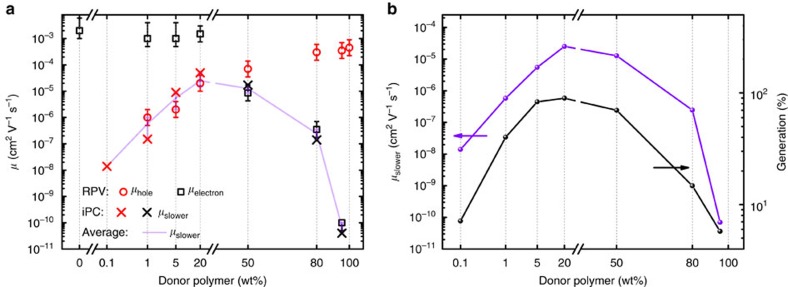
Carrier mobilities and photogeneration yields of PCDTBT:PC70BM blends. (**a**) Electron/hole mobilities for different donor (PCDTBT) fractions as obtained from two different techniques: RPV and iPC. Low-donor blends exhibit high electron and low hole mobilities, while low-acceptor blends exhibit high hole and low electron mobilities. Error bars are determined from the uncertainty in the carrier arrival at the electrodes (see Methods). (**b**) The slower carrier mobility and generation yield follow a similar trend as a function of the blend composition.

Figure 2 shows good agreement between the mobilities obtained by the two techniques. Increasing the donor content from 0.1 to 20% considerably increases the slower carrier (hole) mobility, where we also observe a peak in the charge collection efficiency (Supplementary Fig. 10). Further increasing the donor fraction decreases the electron mobility and electrons become the slower carriers, similar to previous findings^40^. Therefore, we observe a switch between electrons and holes as the slower carrier between 20 wt% to 50 wt% of donor polymer. Across all blend ratios, the slower carrier mobility changes by more than five orders of magnitude from ∼1 × 10^−10^ to ∼3 × 10^−5 ^cm^2 ^V^−1 ^s^−1^ in the 95 wt% and the 20 wt% donor devices, respectively. In contrast, the faster carrier mobility is relatively constant (around 1 × 10^−4 ^cm^2 ^V^−1 ^s^−1^ to 2 × 10^−3 ^cm^2^) and is controlled by the majority phase, that is PCDTBT (PC70BM) for high (low) donor content blends.

Figure 2 also shows that the slower carrier mobility and the generation efficiency follow a similar trend as a function of the blend composition, which indicates that the generation efficiency does not depend on the faster carrier as predicted by Braun's theory. The results for PTB7:PC70BM are shown in Supplementary Fig. 11 and confirm this critical observation.

## Discussion

**Figure 3:**
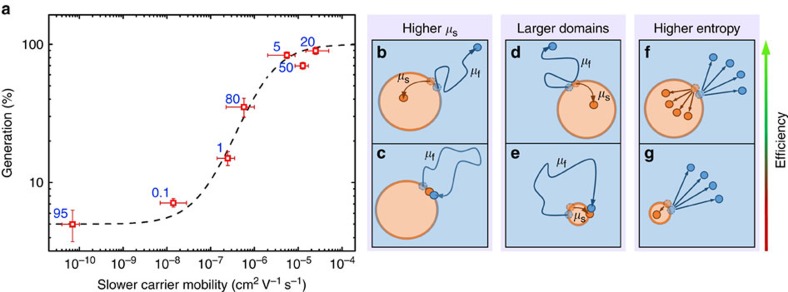
Slower carriers limit charge transfer state dissociation and plausible explanations. (**a**) Generation yields versus slower carrier mobility—either holes or electrons depending on the donor content (the wt% is marked by the numbers). The dashed line is the prediction of Braun's theory, assuming an offset in the generation yield at low mobilities and that the CT state dissociation rate is determined by the slower carrier mobility and not the sum of both mobilities. The *x*-error is the range of the slower carrier mobility as obtained from two independent techniques (iPC and RPV), and the *y*-error bar is the first s.d. of the constant internal quantum efficiency regime (as described in Fig. 1). (**b**,**c**) Increasing the mobility of the slower carrier (orange dot in the dilute phase) enables it to leave the interface, which temporally protects the CT state from recombination and allows the faster carrier (blue dot in the majority phase) to escape. (**d**,**e**) Larger domains of the dilute phase allow the slower carriers to travel further away from the interface, also protecting the CT state from recombination. (**f**,**g**) The entropic contribution to dissociation is maximized in systems where both carriers are mobile because the density of states of two separated charges is vastly larger than the density of states where only one charge is mobile.

These experimental results indicate an important and counter-conventional view: slower carriers—and not the faster ones—play the decisive role in the dissociation of CT states. To gain further insights into the underlying mechanism, we plot in Fig. 3a the measured generation efficiencies against the slower carrier mobilities and compare the observed trend with the CT state dissociation efficiency from Braun's model (dashed line) *η*_CT_=*k*_d_/(*k*_d_+*k*_f_), but where the dissociation rate constant *k*_d_ is assumed proportional to *μ*_s_, and not *μ*_f_+*μ*_s_. The CT state decay rate constant *k*_f_ is fitted to the data, assuming it to be independent of the charge carrier mobilities^9^, which is also consistent with the roughly constant decay times observed in the transient absorption signals shown in Fig. 1b. The match highlights the dominant role of the slower carriers on the dissociation of CT states. We now examine the potential physical mechanisms underlying this important correspondence.

Higher mobility allows the slower carrier to leave the interface and escape recombination: We consider a system with very imbalanced charge transport where the electron and hole are also separated by an interface between different domains, as illustrated in Fig. 3b. Because the faster carrier can escape but also return to the interface substantially quicker than the slower carrier, the motion of the slower carrier cannot be neglected. If the slower carrier is so slow as to be effectively immobile, it remains at the interface and is liable to recombine with the faster carrier (Fig. 3c), whose random walk will be biased towards the slower carrier by their Coulombic attraction. In contrast, if the slower carrier is mobile, it will be able to leave the interface, even if only by a few hops (Fig. 3b). Doing so can temporarily protect the CT state from recombination because the faster carrier cannot enter the slower carrier phase. This increases the likelihood of escape for the faster carrier. It is important to note that the faster carrier can still escape even if the slower carrier is immobile. Pure faster carrier escape can explain why the generation does not decrease to zero at very low slower carrier mobilities, but reaches a plateau of 5% in PCDTBT:PC70BM (Fig. 3a) and 14% in PTB7:PC70BM blends (Supplementary Fig. 11b).

We note that the local mobility in a nm regime (roughly 5–10 nm, which is relevant for CT state separation) might differ from the measured bulk mobility; however, if both mobilities scale in the same way with the blend ratio composition, this mechanism offers a possible explanation of our experimental results.

Larger domains allow the slower carrier to leave the interface—protecting the CT state from recombination: As the fraction of the dilute phase is increased, its domains grow. Larger domains will decrease the recombination rate and thereby increase the dissociation probability of CT states as shown in ref. 25 and illustrated in Fig. 3d. Using Monte Carlo simulations^16^, it has recently been shown that in the limiting case of very small domain sizes the recombination rate will be dependent on the faster carrier (Langevin; Fig. 3e), while the Blom–Koster rate is applicable in the limiting case of a bilayer (if the faster carrier reaches the interface first). The transition to a Langevin system occurs if the domain size approaches ∼5 nm. These theoretical predictions can qualitatively explain our recombination rate measurements, where we find that *k*_r_ scales, in both systems, with the sum of the mobilities (*γ*∼1) in low donor and acceptor blends and becomes most ‘non-Langevin' in the most efficient blends (*γ*∼25 to 33; Supplementary Fig. 9). It is interesting to note that, besides encounter-limited recombination, a recent study also suggests that a large CT state re-dissociation rate constant after free carrier encounters (relative to *k*_f_) could contribute to the suppression of the Langevin recombination coefficient in efficient devices^41^.

Although both properties—a high slower carrier mobility and a sufficiently large domain size—are expected to increase the ability of the slower carrier to leave the interface, their relative contribution to the likelihood of a successful separation event may not follow the same trend as a function of blend ratio composition. For example, in PTB7:PC70BM blends with 10 wt%, 25 wt% and 45 wt% PTB7 we find a relatively constant high slower carrier mobility, while the generation efficiency decreases as the PTB7 concentration is reduced below 45 wt% (Supplementary Figs 5 and 11). Our recombination coefficient measurements suggest that CT states in low donor PTB7:PC70BM blends are less protected from recombination, which could explain the observed drop in the generation efficiency. However, further work is required to disentangle the effect of the domain size and slower carrier mobility.

Entropy favours the simultaneous dissociation of both carriers: Lastly, changes in the donor and acceptor domain sizes will also change the entropic contribution to the dissociation, as illustrated in Figs 3f and g. A growing body of evidence suggests that entropy facilitates charge separation because of the expansion of the number of available states as the carriers diffuse away from the interface^42^,^43^,^44^,^45^. The free energy of dissociation is given by


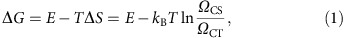


where *Ω*_CS_ and *Ω*_CT_ are the numbers of accessible CS and CT states, respectively, *E* is the CT state binding energy, *k*_B_ the Boltzmann constant and *T* the temperature. In the extreme case of very imbalanced donor:acceptor concentrations, where only the faster carrier has ways to escape, *Ω*_CS_ would equal *Ω*_f_—the number of states accessible to the faster carrier (Fig. 3g). By contrast, in balanced donor:acceptor systems, where the slower carrier is also able to leave the interface, the factor *Ω*_CS_ will be proportional to *Ω*_f_ *Ω*_s_, which greatly increases the entropic contribution to charge separation (Fig. 3f). This means that the entropic drive is stronger in blends where both carriers are mobile and are able to leave the interface, reducing the free-energy barrier for dissociation. The entropic contribution to the dissociation rate thereby further supports our fundamental assertion that the dissociation is largely dependent on the slower carrier.

In conclusion, we have experimentally studied the impact of the charge carrier mobilities on the dissociation yield of CT states in organic semiconductor blends using the archetypal BHJ organic solar cell architecture. Our electrical transport results, which are supported by independent TAS measurements, do not agree with the common view that charge separation occurs when the faster carrier makes a rapid escape, and underscore the failure of a simple Braun model, which is based on the dissociation rate being proportional to the sum of the mobilities. Our data strongly suggest that it is largely the ability of the slower charge carriers to leave the donor:acceptor interface that dictates the efficiency of CT state dissociation. Possible mechanisms that enable the slower carriers to leave the interface are as follows: a high enough mobility, a sufficiently large domain size and enough conduction pathways that lower the Coulomb barrier for dissociation because of entropic effects. Our findings are important as they shed new insight into the fundamental physics of organic semiconductors, and also provide new structure–property strategies for optimizing charge generation in excitonic light-harvesting systems. Namely, they underscore the need for high mobilities to maximize not only charge collection but also charge generation, and further demonstrate the role of a balanced donor:acceptor blend ratio to maintain the mobility and domain size of slower carriers and a high system entropy.

## Methods

### Device preparation

Glass substrates with an 80nm indium tin oxide (purchased from Kintec) layer were cleaned by sonicating in sequence with Alconox, deionized water, acetone and 2-propanol for 5 min. Subsequently, the substrates were coated with 30 nm of poly(3,4-ethylenedioxythiophene):poly(styrene sulfonate) (PEDOT:PSS; Baytron P VPAl4083). PCDTBT (SJPC, Canada, Mw=122,200 g mol^−1^, polydispersity index (PDI)=5.4) and PC70BM (American Dye Source Inc., Canada, Mw=1,032 g mol^−1^) active layer blends were fabricated by first preparing solutions of PCDTBT (30 mg) in 1,2-dichlorobenzene (5 ml) and PC70BM (120 mg) in chlorobenzene (5 ml). The solutions were then mixed in an appropriate ratio to obtain the specified blend ratio compositions. The solutions were spin-coated on the substrates for 90 s, while the spin speed was varied to achieve the same target-active layer thickness (75 nm) for each blend. Blends of PTB7 (1-Material, Mw=97.5 kDa, PDI=2.1) and PC70BM were prepared by first separately dissolving PTB7 (90 mg) and PC70BM (120 mg) in a mixture of 1,2-dichlorobenzene and chlorobenzene (50:50%, 5 ml). The solutions were again spin-coated on the substrates for 90 s, while the spin speed was varied to achieve a similar target-active layer thickness (125–150 nm) for each blend. The active layer thicknesses were measured with a DekTak 150 profilometer. All devices were completed by vacuum evaporation of 1.2 nm of samarium, followed by 75 nm of aluminium under a 10^−6^-mbar vacuum. The device area was 0.2 cm^2^. The device fabrication took place within a glove box with <1 p.p.m. O_2_ and H_2_O, and *JV* and EQE measurements were also performed inside a glove box. Subsequently, the devices were encapsulated for the iPC, TAS, RPV and charge extraction measurements in the dark using linearly increasing voltage (dark-CELIV). Reflectometry for calculating the IQE was measured on duplicate devices with a device area of 6.25 cm^2^ using an integrating sphere and a PV Measurements Inc. QEX7 system.

### Current density–voltage characteristics

*JV* curves were obtained in a two-wire source-sense configuration, and an illumination mask was used to prevent photocurrent collection from outside of the active area. An Abet Class AAA solar simulator was used as the illumination source, providing 100 mW cm^−2^ of AM1.5G light. The exact illumination intensity was used for efficiency calculations, and the simulator was calibrated with a standard traceable photodiode from the National Renewable Energy Laboratory.

### Light-intensity-dependent measurements

Steady-state intensity-dependent photocurrent measurements were performed with a 532-nm continuous wave laser (Ningbo Lasever Inc.), providing a power of 1 W. Optical filters (ThorLabs) were used to attenuate the laser power, and the photocurrent transients were recorded with an Agilent semiconductor device analyser (B1500A). Each measured data point corresponded to a steady-state photocurrent measurement at the respective incident laser power, which was simultaneously measured with a Silicon photodetector to improve the accuracy of the measurement. iPC was repeated on several pixels for each blend composition. The EQE was obtained from the ratio of the photocurrent and the laser power. The EQE values obtained from the iPC measurement were compared with the EQE spectra that were measured using a PV Measurements Inc. QEX7 system. The IQE was subsequently calculated from the ratio of the EQE and the active layer absorption. The latter was obtained from specular reflectance spectra and simulated absorption by the non-active layers using a code developed by van de Lagemaat and co-workers^46^ from the National Renewable Energy Laboratory. More details on how the active layer absorptions are obtained are provided in Supplementary Fig. 4.

### Repetitive and resistance-dependent photovoltage

RPV for mobility, recombination coefficient and trapping measurements was recorded with an oscilloscope (LeCroy WaveRunner 6200A) with different external load resistances (*R*_Load_), while a delay generator (Stanford Research Systems DG535) was used to trigger a function generator (Agilent 33250A) and a pulsed Nd:Yag laser (Brio Quantel) with a pulse length of 10 ns. An excitation wavelength of 532 nm was used to generate the charge carriers, while neutral optical density filters were used to attenuate the ∼50mJ energy output. For the RPV mobility measurements, low laser pulse intensities (resulting in a photovoltage close to 100 mV at a load resistance *R*_Load_ of 1 MΩ) were used to avoid space charge effects and to maintain quasi short-circuit conditions^31^. However, the transients were also measured under various applied biases. To estimate the recombination coefficients, the extracted charge (*Q*_ext_) was calculated at different load resistances by integrating the photovoltage transients measured at highest pulse intensities that saturate the photovoltage. Repetitive photovoltage transients for charge-trapping measurements and very low mobility detection (on the order of 10^−10 ^cm^2 ^V^−1 ^s^−1^) were recorded at *R*_Load_=1 MΩ and different laser repetition rates (2–20 Hz). Summaries of these measurement techniques are provided in the figure captions of Supplementary Figs 7–9, respectively. The error bars in the mobility values as measured by RPV indicate the uncertainty of the carrier transit times. The uncertainty of the transit time was approximated from the range at which the photovoltage signals deviate and saturate to tangents fitted to the rise and plateau regions of the photovoltage transients (as plotted in Supplementary Figs 7 and 8).

### TAS

Femtosecond TAS was carried out using a commercially available transient absorption spectrometer, HELIOS (Ultrafast Systems). Samples were excited with a pulse train generated with an optical parametric amplifier, TOPAS (Light Conversion). Both the spectrometer and the parametric amplifier were seeded with an 800nm, <100fs pulse at 1 KHz generated with a Solstice Ti:Sapphire regenerative amplifier (Newport Ltd). Changes in the optical density of the films induced by the laser excitation were followed with a second broadband pulse (830–1,450 nm) generated in a sapphire crystal. The HELIOS transient absorption spectrometer was used for recording the dynamics of the transient absorption spectra up to 2.7 ns with an average 200-fs instrument response function. Measurements were performed on the active layer next to the top electrode of the same devices as used for the electrical measurements (structure: glass/indium tin oxide/PEDOT:PSS/Active Layer). Samples were excited at 560 nm with a fluence of 500 nJ cm^−2^. The low fluence ensured the absence of second-order recombination processes. The decay dynamics were analysed corresponding to the polymer exciton (1,300 nm) and positive polaron (cation) absorption (1,000 nm). Global analyses of the data were carried out using the programme OriginLab. Repetitive TAS signals have been averaged 10 times and measurements repeated twice.

### Data availability

All relevant data are available from the authors.

## Additional information

**How to cite this article**: Stolterfoht, M. *et al*. Slower carriers limit charge generation in organic semiconductor light-harvesting systems. *Nat. Commun.* 7:11944 doi: 10.1038/ncomms11944 (2016).

## Supplementary Material

Supplementary Information Supplementary Figures 1-11, Supplementary Tables 1-3 and Supplementary References

Peer review file
