# Supplementary material for: Slower carriers limit charge generation in organic semiconductor light-harvesting systems
Source: Nat Commun. 2016 Jun 21;7:11944. doi: 10.1038/ncomms11944 (PMC4919513; doi:10.1038/ncomms11944)
Supplement: Supplementary Information — Supplementary Figures 1-11, Supplementary Tables 1-3 and Supplementary References [file ncomms11944-s1.pdf]

Supplementary Figures

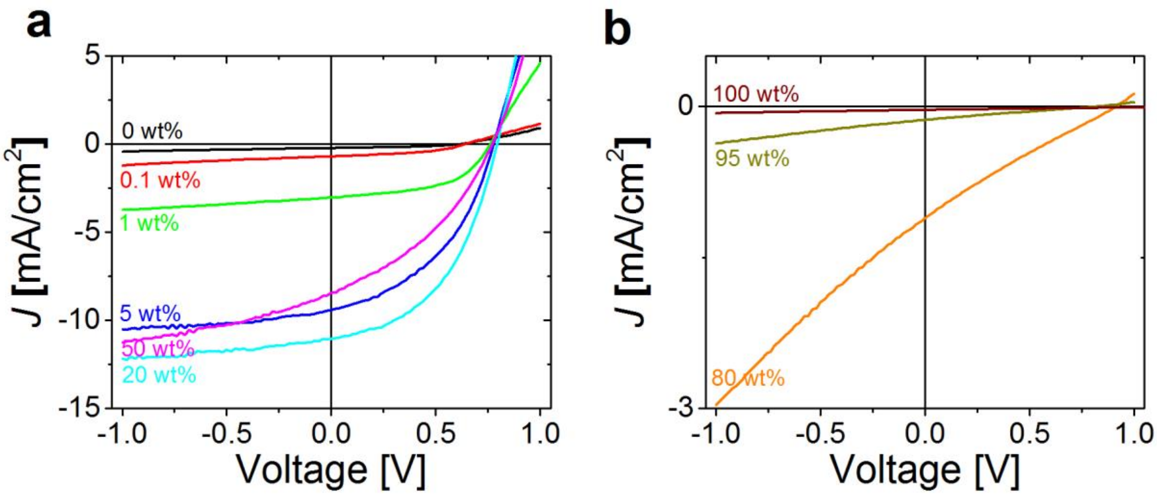

Supplementary Figure 1. JV-curves of PCDTBT:PC70BM blends. Representative white light current density-voltage (JV) characteristics obtained under standard AM 1.5G illumination for **(a)** low donor PCDTBT:PC70BM blends and **(b)** high donor PCDTBT:PC70BM blends. The amount of PCDTBT in each blend in wt% is marked.

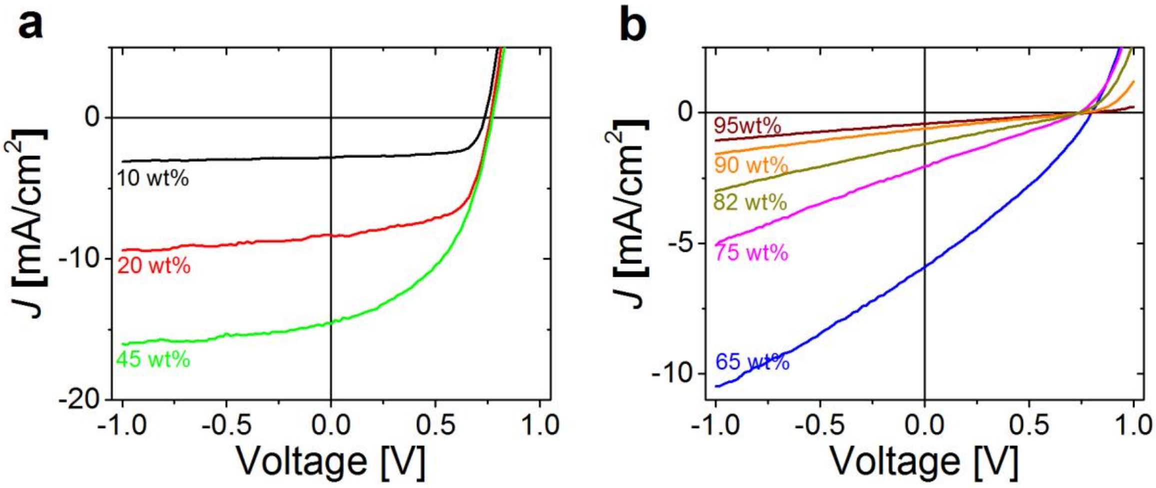

Supplementary Figure 2. JV-curves of PTB7:PC70BM blends. Representative white light current density-voltage (JV) characteristics obtained under standard AM 1.5G illumination for **(a)** low donor PTB7:PC70BM blends and **(b)** high donor PTB7:PC70BM blends. The amount of PTB7 in each blend in wt% is marked.

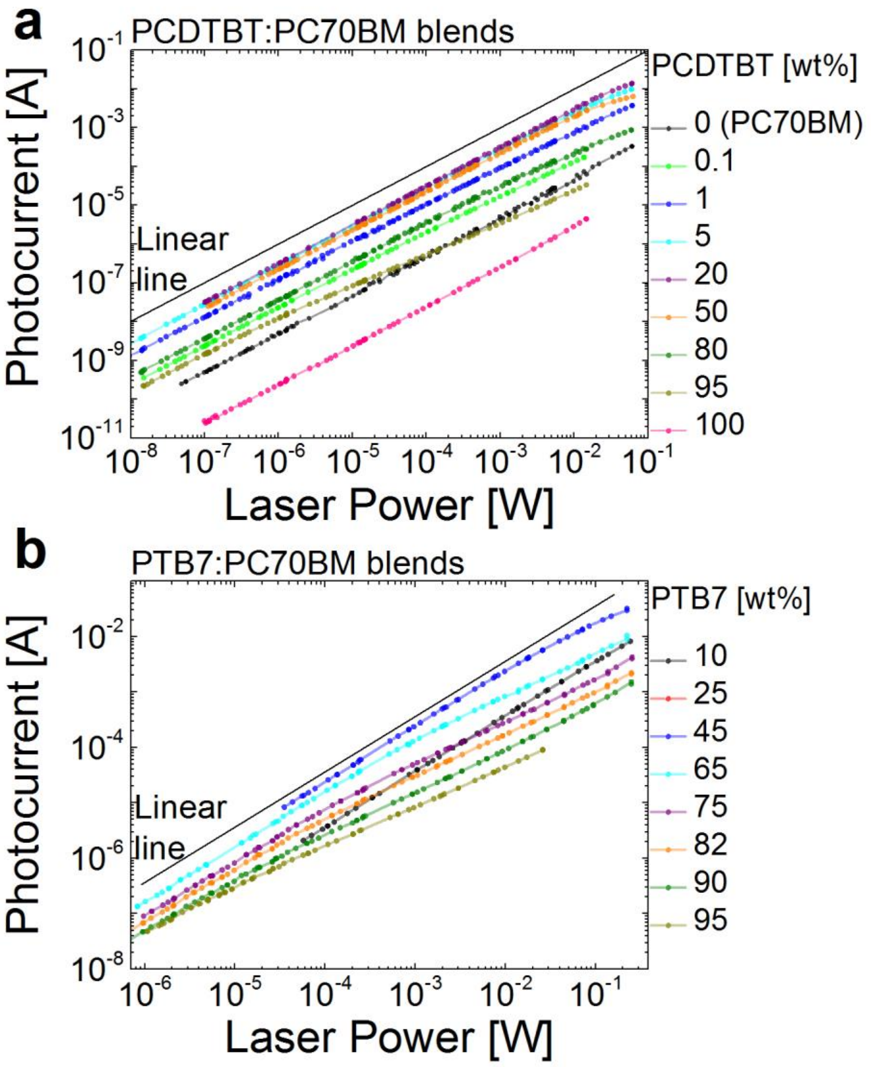

Supplementary Figure 3. Intensity dependent photocurrent of all studied devices. The laser power dependent photocurrent of each (**a**) PCDTBT:PC70BM blend and (**b**) PTB7:PC70BM solar cells with varying blend ratios. This data was used to calculate the photocurrent dependent internal quantum efficiencies (IQE, **Figure 1a**) and to estimate the slower carrier mobility, as described in the main text. The IQE is obtained from the ratio of photocurrent and the laser power and the active layer absorption (**Supplementary Figure 4**).

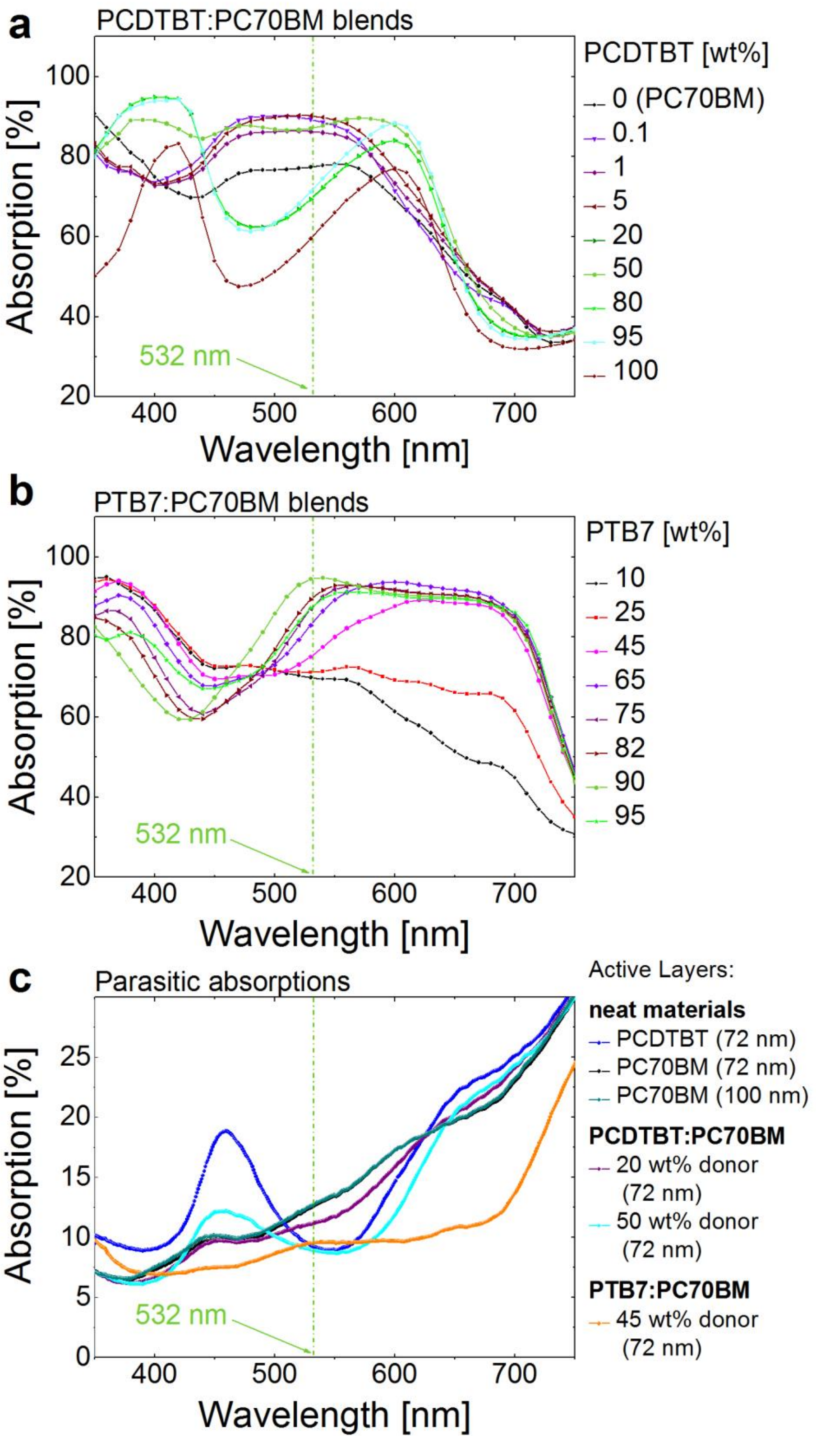

Supplementary Figure 4. Device and parasitic absorption spectra of all studied devices. The absorption spectra of the (**a**) PCDTBT:PC70BM and (**b**) PTB7:PC70BM devices. The absorption spectra $A(\lambda)$ were obtained by measuring the reflectance spectra $R(\lambda)$ on the operational devices *via* $1 - R(\lambda)$ using a universal reflectance attachment on a Perkin-Elmer Lambda 950 spectrophotometer accurately baselined with a reference glass slab. The device absorption at 532 nm minus the parasitic

absorptions in non-active layers is used to calculate the intensity dependent internal quantum efficiencies (**Figure 1a** and **Supplementary Figure 5)**. (**c**) Simulated parasitic absorptions in non-active layers (indium tin oxide, PEDOT, aluminium) of neat PCDTBT and PC70BM diodes; two PCDTBT:PC70BM blends with 20 wt% and 50 wt% PCDTBT; as well as a PTB7:PC70BM blend with 45 wt% donor (the active layer thickness is specified in the graph). It is demonstrated that diodes of neat materials and blends exhibit very similar parasitic absorptions at 532 nm excitation ranging from 9% (in PCDTBT:PC70BM 1:1) to 12.7% (in PC70BM). The parasitic loss at 532 nm in diodes with low donor active layers (of which the optical constants n and k were not available) was approximated from the loss in a PC70BM-only diode. Likewise, the parasitic loss in high donor PCDTBT:PC70BM blends was approximated from the loss in PCDTBT-only diodes. For high donor PTB7:PC70BM blends we assumed a similar parasitic loss (at 532 nm) as in the 45 wt% PTB7:PC70BM blend. To simulate the optical field distribution and absorption of the layered stack a computational code was used that is based on the transfer matrix method [1] and developed by van de Lagemaat et al. from the National Renewable Energy Laboratory (NREL). Additional details concerning the ellipsometry and optical model are presented in [2].

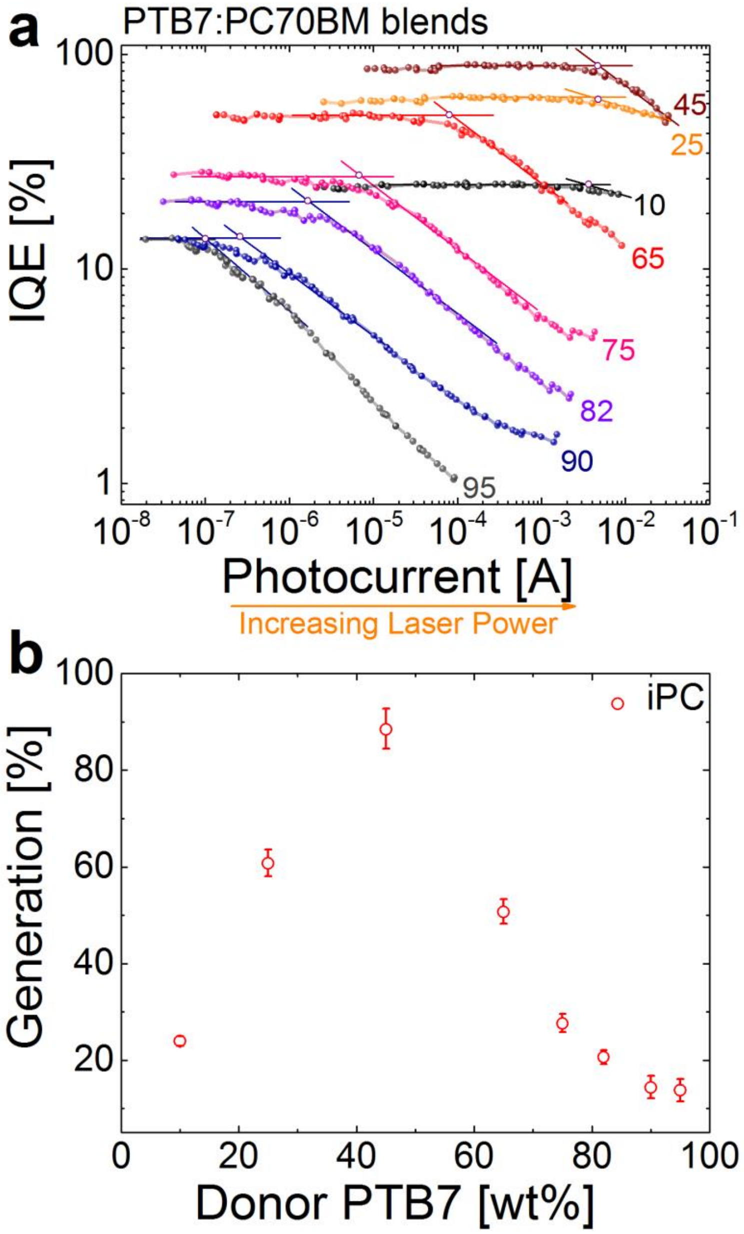

Supplementary Figure 5. Photocurrent dependent IQE and generation yields of PTB7:PC70BM blends. (**a**) Internal quantum efficiencies (IQEs) of PTBT:PC70BM devices with varying blend ratios plotted as a function of the photocurrent of each device in analogy to **Figure 1** in the main text. (**b**) The generation yield estimated from the constant IQE values of each blend as a function of the PTB7 fraction. The generation efficiency peak at a polymer fraction of 45 (wt%) in line with the short-circuit current density and device performance (**Supplementary Figure 2**). iPC error bars are determined from the first standard deviation of the constant IQE regime, considering a relative variation of 5% of the active layer absorption.

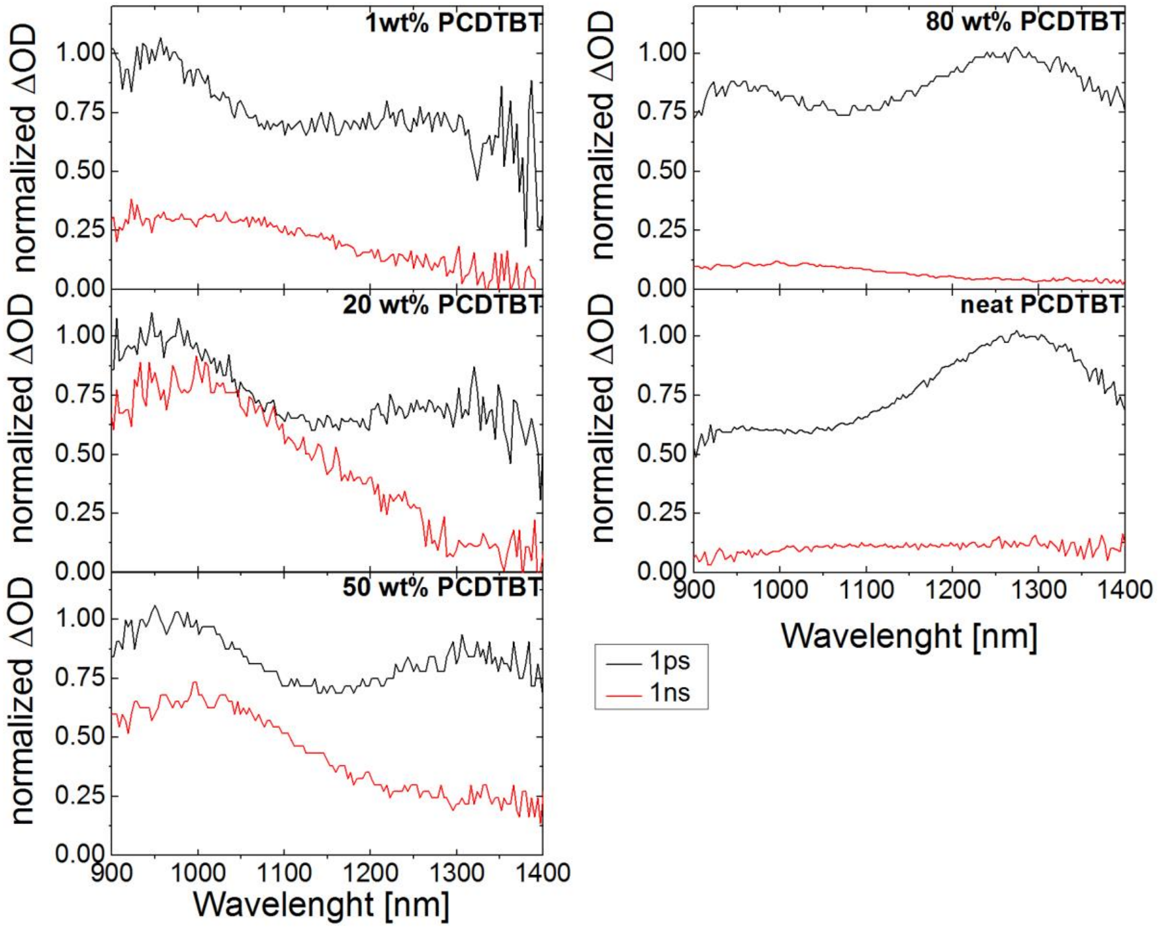

Supplementary Figure 6. Near infrared transient absorption spectra of PCDTBT:PC70BM blends. Femtosecond transient absorption spectra for different PCDTBT:PC70BM blends recorded 1 ps after excitation (black curves) and 1 ns after excitation (red curves). An excitation wavelength of 560 nm was used and a low laser fluence of 500 nJ cm^{-2}. The NIR transient absorption for the neat PCDTBT has been assigned previously to photoinduced absorption from singlet excitons, peaking at 1400 nm [3]. For blends, the transient absorption spectra at early times (200 fs) exhibit a broad absorption peaking at ≈1300 nm similar to that of the neat PCDTBT. However, for these blend systems, this spectrum rapidly evolves to a narrower photoinduced absorption peaking at 1000 nm, assigned to photoinduced charge separation from polymer singlet excitons to polarons, which is also consistent with previous assignments [3]. The dynamics at 1300 nm and 1000 nm were measured and modelled from the femto- to the microsecond time range and include the key phases of the carrier dynamics.

The *exciton decay dynamics* for the different compositions were monitored at the PCDTBT exciton photoinduced absorption maximum of 1300 nm. In all cases, the decay dynamics are dominated by a monoexponential decay phase assigned to photoinduced polymer exciton recombination, with the small residual signal (at 1ns) being assigned to triplet (for neat PCDTBT) and polaron absorption for blends with PCBM. It is apparent that all systems exhibit similar exciton decay dynamics, with similar time constants for the different blend devices (**Supplementary Table 3**). The strong polymer emission quenching in all blend films is clearly indicative of mixing of polymer and acceptor species on length scales less than the polymer exciton diffusion length and indicates efficient polymer exciton separation. The kinetics of the 1000 nm band are fitted to an exponential decay. The decay time of all of the blends varies between several 100 ps to 3 ns, which we attribute to geminate recombination

of interfacial CT states. At longer times (data not shown), devices show approximately a power law decay of the polaron absorption, assigned to polaron recombination to ground. These dynamics are intensity dependent, accelerating as the excitation density is increased, indicating they should be assigned to non-geminate recombination of dissociated polarons rather than bound polaron pairs.

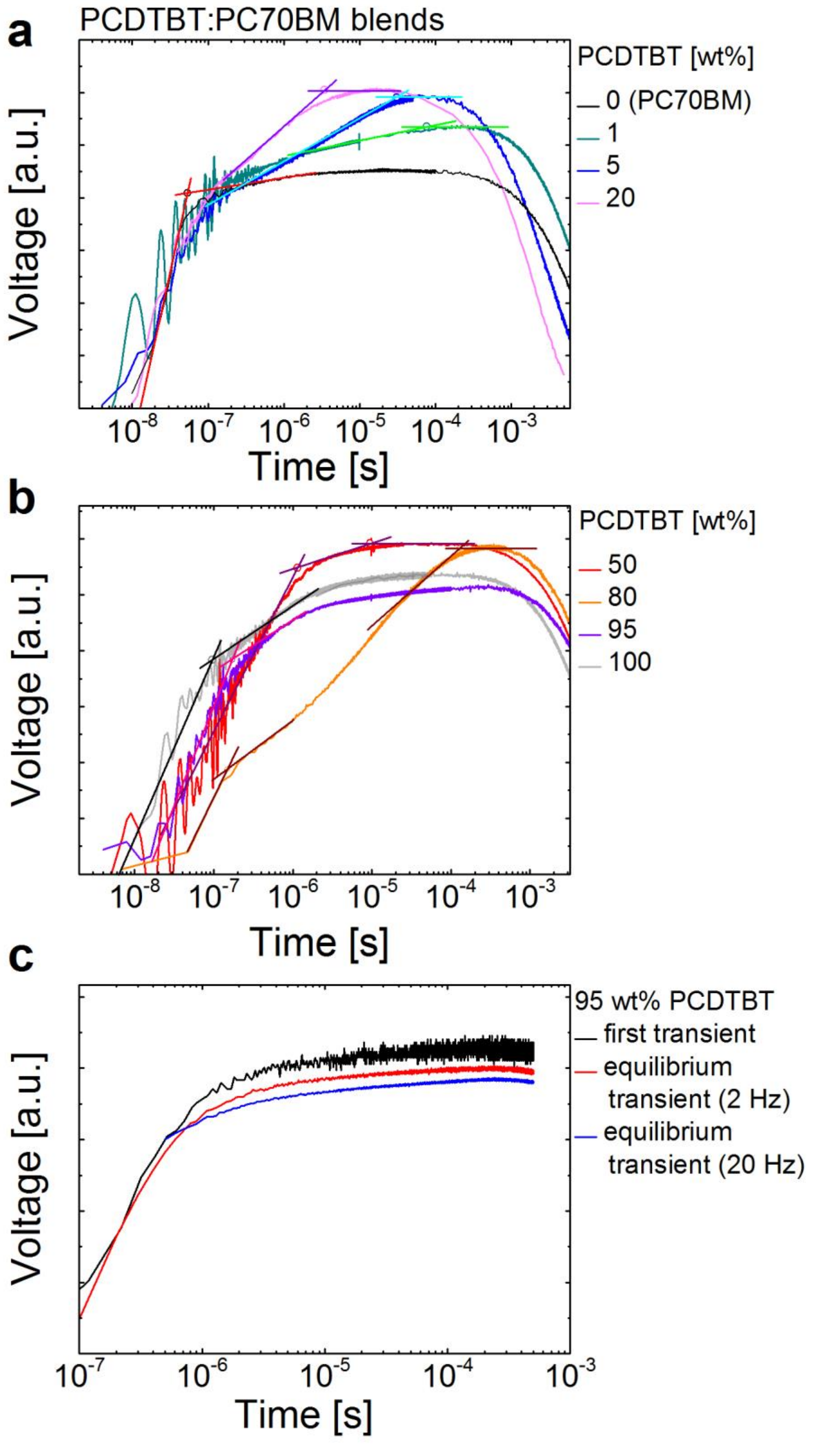

Supplementary Figure 7. Transient and repetitive photovoltage signals of PCDTBT:PC70BM blends. (**a, b**) Photovoltage transients of PCDTBT:PC70BM solar cells with varying blend ratios to estimate the charge carrier mobilities following the methodology introduced in [5]. Transients are measured at a high load resistance (R_{LOAD}) of 1 MΩ, without an external voltage applied. A low-intensity laser pulse (resulting photovoltage << open circuit voltage) is used to prevent the redistribution of the internal

electric field. Therefore, the charge carriers are extracted under quasi short-circuit conditions even at a large load resistance. Because, the ability of the external circuit to discharge the electrodes (given by the resistance-capacitance product *RC*) is greatly reduced, the photovoltage peaks if all carriers are extracted from the film, which marks the transit time. When the mobilities of electrons and holes are imbalanced, then two shoulders are observed in the transients, corresponding to the arrival of faster and slower carriers. It is also required to ensure the saturation of the maximum photovoltage with increasing R_{Load}, otherwise the transit time will be underestimated. The photovoltage transients of the neat materials are used to identify which shoulder corresponds to electrons and holes. (**a**) PC70BM-only diode: the sharp voltage rise in the *black* transient followed by a voltage saturation demonstrates the quick transit of electrons. The transit time is estimated from the intersection of the lines (~50 ns). Upon adding 1 wt% to 20 wt% of PCDTBT, we observe the development of a second photovoltage shoulder that reveals the transport of holes in the PCDTBT domains. It can be seen that the hole mobility increases significantly when the PCDTBT concentration is increased from 1 wt% to 20 wt%, while the electron mobility remains essentially constant (within the uncertainty of the measurement). (**b**) Upon further increasing the PCDTBT content to 50 wt% (*red transient*) the pronounced first shoulder (that is observed for PC70BM-only and low donor blends) vanishes, which suggests a decreasing percolation of PC70BM domains and consequently a reduction of the electron mobility. This results in a more balanced electron and hole transport. We assume the hole mobility in the blend has increased compared to the 20 wt% PCDTBT:PC70BM blend, which means that the slower carriers are the electrons in this blend. At a PCDTBT concentration of 80 wt% (*orange transient*) we again observe two photovoltage shoulders. A small shoulder is observed at (~150ns) that is attributed to holes and a pronounced shoulder at long timescales (> 100µs), which is assigned to a low effective electron mobility due to insufficient PC70BM percolation. At 95 wt% PCDTBT (*violet transient*), the first shoulder corresponding to the hole transport increases in magnitude (compared to the 80 wt% blend) and closely approaches the transport that is observed in a neat PCDTBT diode (*grey transient*). The second shoulder in the 95 wt% PCDTBT blends cannot be resolved with the maximum available R_{Load} of 1 MΩ, because the electron transit time is much longer than the external discharge (*RC*) time. (**c**) To estimate the electron mobility in the 95 wt% PCDTBT blend we used the repetitive photovoltage [6] technique, which allows us to assess slow charge transport processes. In this experiment, the transients are measured at a repetition rate of 2 Hz (*red transient*) or 20 Hz (*blue transient*) at a R_{Load} of 1 MΩ. The transient of the first laser shot (*black*) is recorded after resting the device in the dark, which allows deeply trapped charges to escape. If the release time of electrons on isolated PC70BM domains is (for example) much longer than 500 ms, then electrons will accumulate upon repetitive laser excitations at 2 Hz. The difference in the magnitude between the "first transient" and the saturated transient after many laser shots reveals the presence of these long-lived trapped carriers because they cause recombination with newly generated carriers. It can be seen that the magnitude of the equilibrium transient increases and approaches the magnitude of the first transient when then repletion rate is lowered from 20 Hz to 2 Hz. This allows one to specify a lower limit for the transit time of electrons of 500 ms. For all other blend ratios we observed identical first and equilibrium transients, because carriers are extracted on much shorter time-scales as shown in (**a**, **b**).

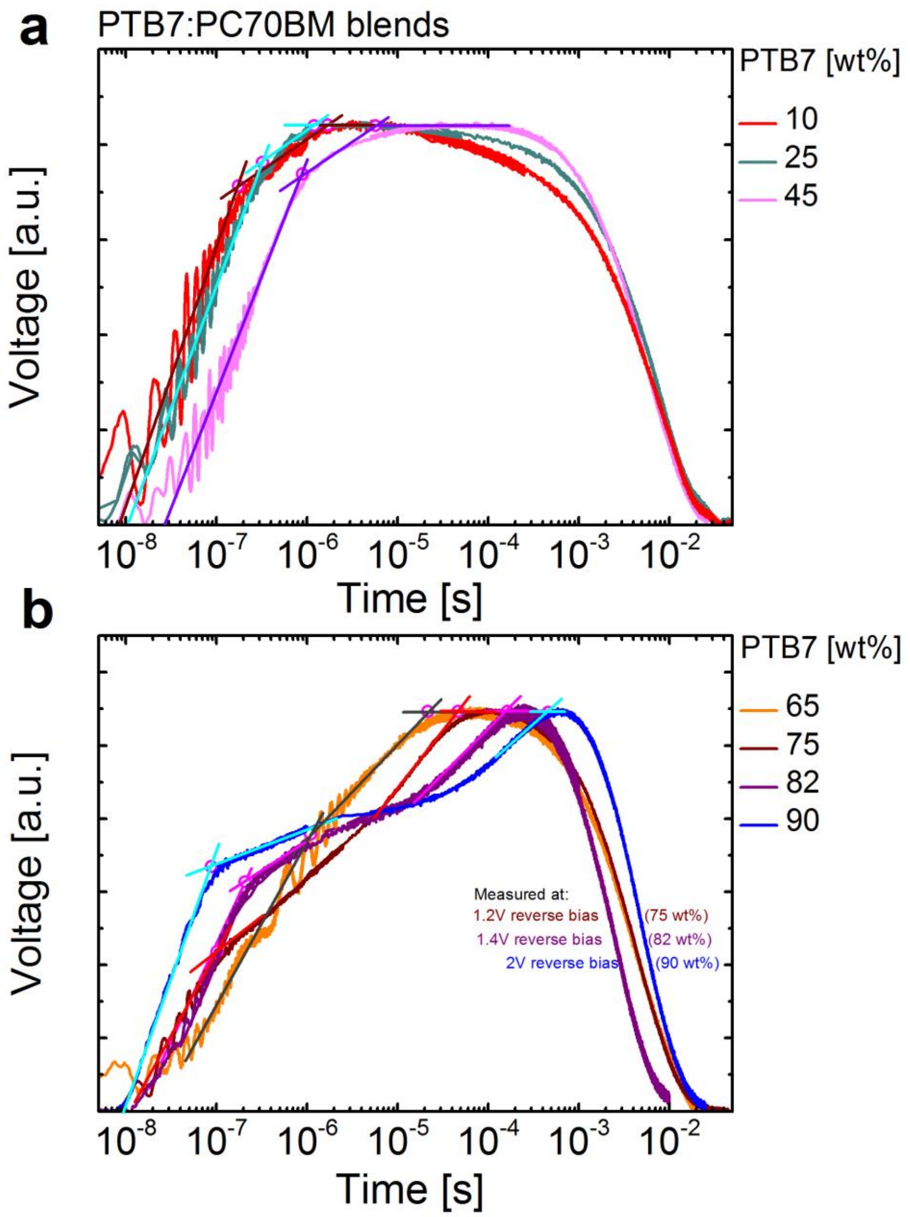

Supplementary Figure 8. Transient photovoltage signals of PTB7:PC70BM blends. Photovoltage transients of PTB7:PC70BM solar cells with varying blend ratios to estimate the charge carrier mobilities in analogy to measurements on PCDTBT:PC70BM (**Supplementary Figure 7**).

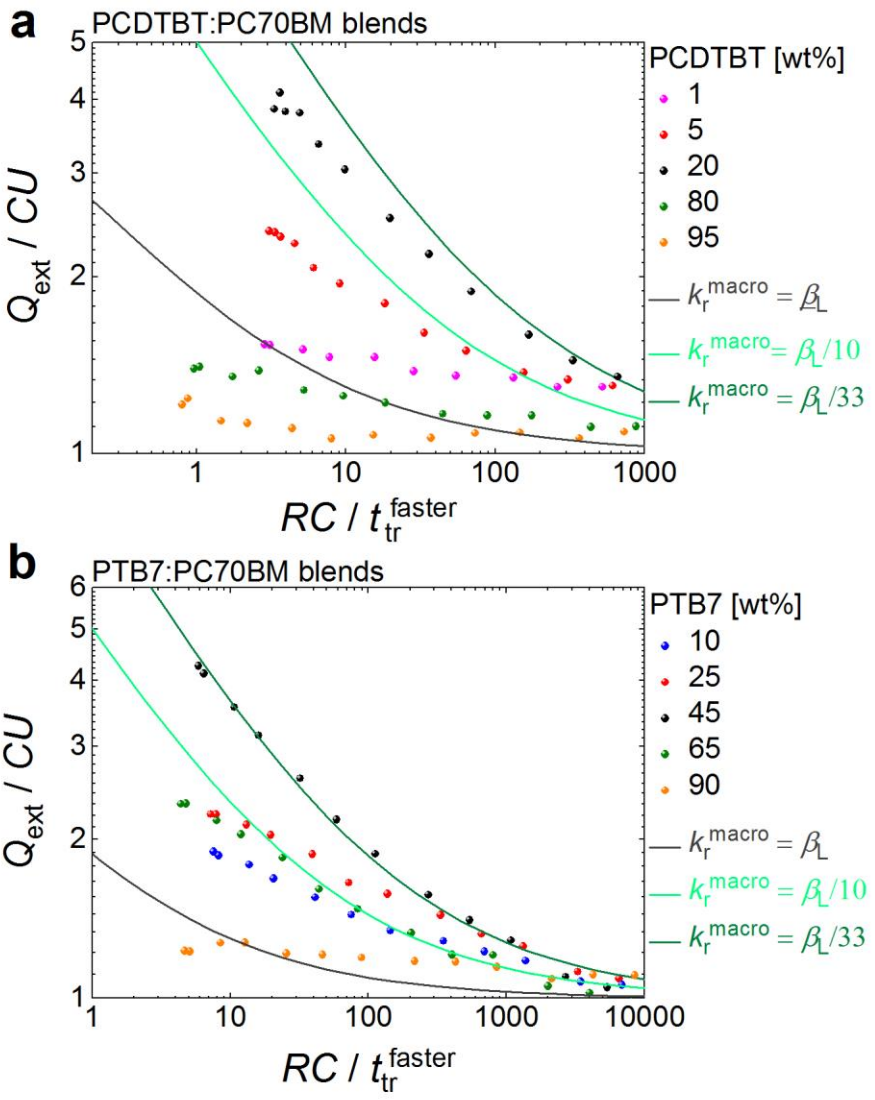

Supplementary Figure 9. Transient extracted charge of all studied devices. The charge extracted (Q_{ext}) for (**a**) PCDTBT:PC70BM blends and (**b**) PTB7:PC70BM blends normalized to their electrode charge CU and plotted as a function of the circuit time constant (the resistance-capacitance product RC) normalized to the faster carrier transit time. Q_{ext} is measured at different load resistances by integrating the photovoltage transient response (similar to those shown in **Supplementary Figure 7**) after a short laser excitation at highest pulse intensities that saturate the photovoltage. The data allows one to estimate the (macroscopic) recombination coefficient k_{r} in relation to the Langevin recombination coefficient (β_{L}) [7]. The application of a high laser fluence excitation immediately screens the electric field inside the film, whereas photogenerated carriers either recombine due to diffusion or get extracted. The load resistance in the circuit is varied which changes the *RC*-time of the system to effectively vary the rates of extraction and recombination of the photogenerated charges. Depending on k_{r} that affects the lifetime of the charges - more or less charges can be extracted. At largest load resistances, where the RC time is much larger than the extraction time and carrier lifetime, Q_{ext} is expected to saturate to CU (the charge capable of being stored on the electrodes). Reducing the RC time via the load resistance allows the extraction of more charge depending on the photocarrier lifetime and the bimolecular recombination coefficient. Using drift-diffusion simulations

allows one to fit k_{r} to the experimental data [7]. (**a**) k_{r} varies in PCDTBT:PC70BM blends from > β_{L} (at 95 wt% PCDTBT loading) to ~$\beta_{\mathrm{L}}/25$ (at 20 wt% PCDTBT loading), although we note that Q_{ext}/CU obtained in PCDTBT:PC70BM blends with very imbalanced donor:acceptor concentrations cannot be fitted to the predictions of numerical simulations. This might be attributed to an insufficient laser power in these blends with low photocarrier generation yields. (**b**) In PTB7:PC70BM blends k_{r} changes from ~β_{L} (in the blend with 90 wt% PTB7) to ~$\beta_{\mathrm{L}}/33$ (in the 45 wt% PTB7:PC70BM blend). We observe in both systems that blends with a high generation efficiency exhibit a low recombination coefficient (at these high laser intensities) and that charges are less protected from recombination in inefficient blends. The results can be explained by an increasing domain size of the dilute phase from low donor (or acceptor) blends to balanced donor:acceptor blends. The results suggest the importance of the slower carrier domain size for protecting the CT state from recombination as discussed in the main text (**Figure 3d, e**). The recombination coefficients were taken into account to estimate the slower carrier mobility from the intensity dependent photocurrent data (**Figure 1a**, **Supplementary Figure 5**) according to [6].

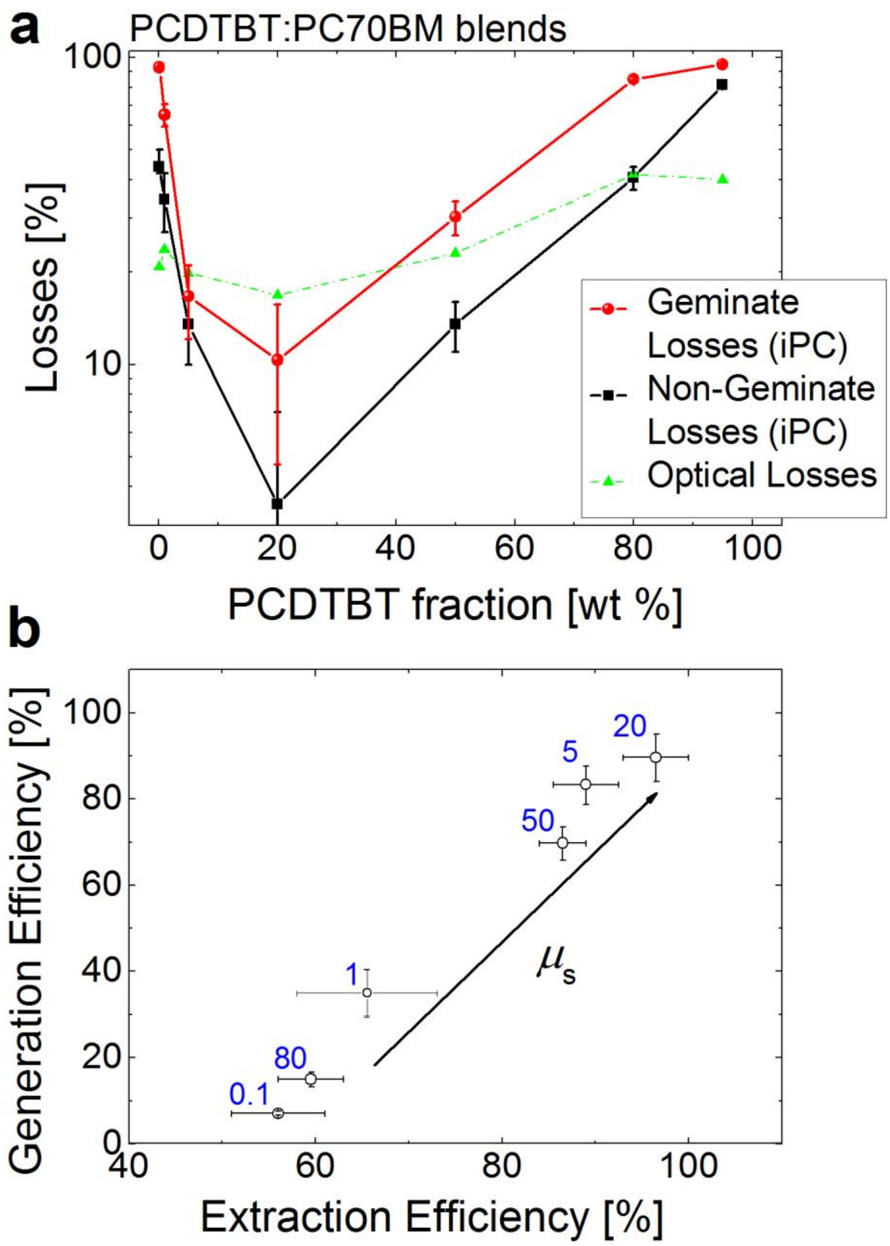

Supplementary Figure 10. Losses in the collection and photogeneration yields of PCDTBT:PC70BM blends. (**a**) Geminate and non-geminate recombination losses under 1-sun equivalent conditions as a function of the donor fraction in the PCDTBT:PC70BM blends determined using the intensity dependent photocurrent technique as described in [6]; as well as optical losses in the active layer. Both geminate and non-geminate losses follow a similar trend and are at a minimum at the optimized composition ratio of 1:4. (**b)** Map of charge generation efficiency versus charge extraction. The dependence of these two efficiencies on the slower carrier manifests as an apparent interdependency between charge generation and extraction. Generation efficiency (and geminate recombination) error bars are determined as explained in **Figure 1**. Extraction efficiency (and non-geminate recombination) error bars are estimated from the range of the ratios of the internal quantum efficiency values at 1-sun and the constant regime, considering two measurements for each blend.

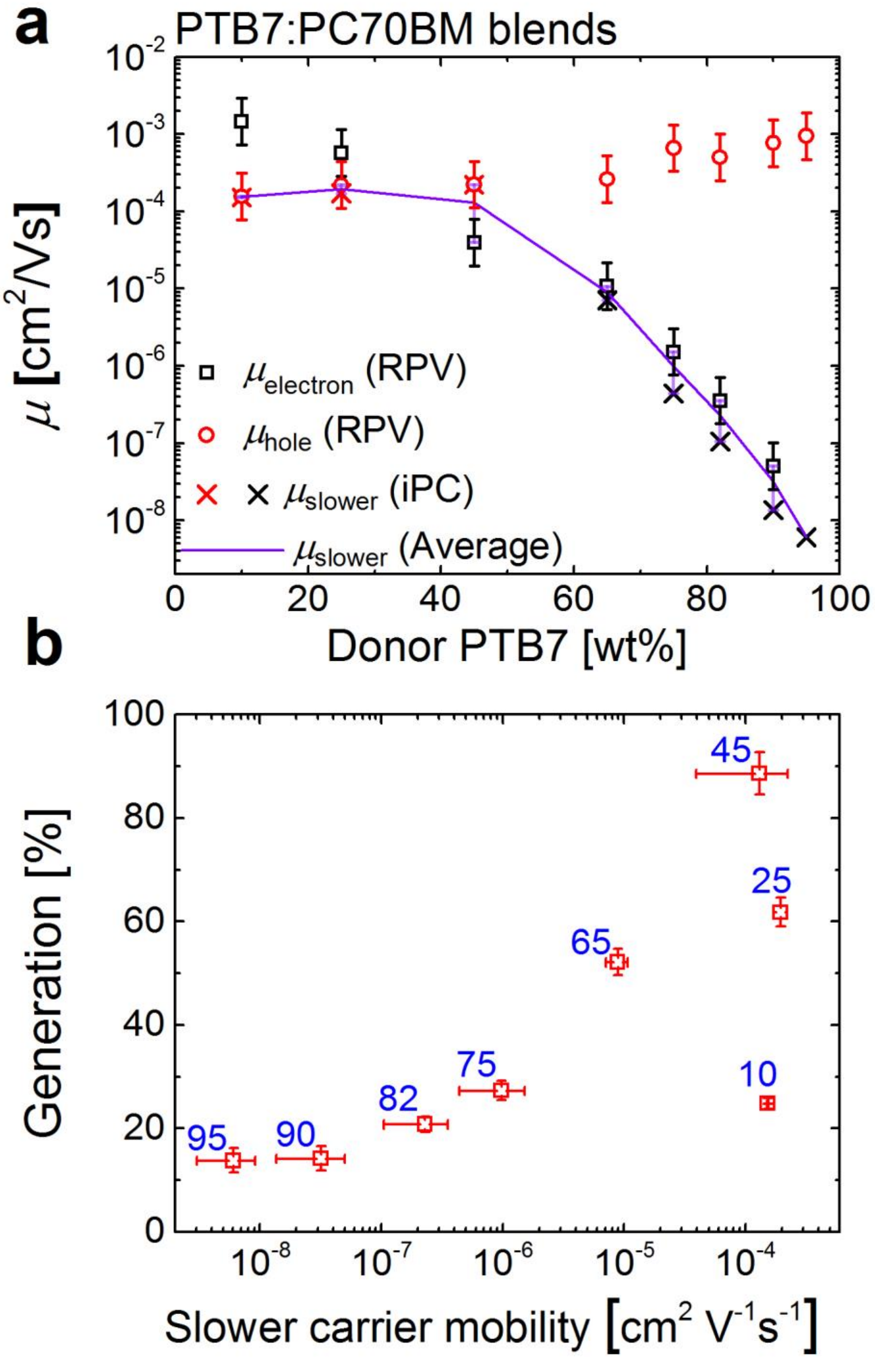

Supplementary Figure 11. Carrier mobilities versus generation yields of PTB7:PC70BM blends. (a) Faster and slower carrier mobilities in the PTB7:PC70BM system as a function of the donor fraction obtained from Resistance dependent Photovoltage (RPV, **Supplementary Figure 8**) and intensity dependent photocurrent (iPC, **Supplementary Figure 5**) measurements, analogous to **Figure 2**. The results are similar to the PCDTBT:PC70BM system. A switch of the slower carrier type is observed for a PTB7 concentration of 45 wt%. The slower carriers are holes at lower concentrations, and electrons

at higher donor concentrations. Very low slower carrier mobilities are observed in high donor blends similar to the high donor PCDTBT:PC70BM blends. However, no sharp drop in the hole mobility is detected as the donor concentration is reduced to 10 wt% (**b**) The generation yields of the PTB7:PC70BM blends as estimated from the intensity dependent IQE data (**Supplementary Figure 5**) as a function of the slower carrier mobility. We observe an increase of the generation efficiency with the slower carrier mobility from the high donor regime until the optimum blend ratio (~45 wt% PTB7), while the generation efficiency drops distinctly at lower donor concentrations (25 wt% and 10 wt%) with essentially constant slower carrier mobilities. The results indicate that a high slower carrier mobility (**Figure 3b, c**) might not be enough for efficient generation and the shape of the donor (acceptor) domains may also significantly influence the dissociation efficiency. In low donor PTB7:PC70BM blends the slower carrier (and therefore the CT state) might not be sufficiently protected from recombination, despite exhibiting a relatively high slower carrier mobility. This argument is supported by the extracted charge measurements (**Supplementary Figure 9**) at highest laser intensities, where we find that the extracted charge decreases with decreasing polymer amount below 45 wt%. The x-error bars are obtained from the difference in the slower carrier value as obtained from the intensity dependent photocurrent measurements (**Supplementary Figure 5**) and the resistance dependent photovoltage measurements (**Supplementary Figure 8**). The y-error bars represent the first standard deviation of the constant IQE regime, considering a relative variation of 5% of the active layer absorption.

Supplementary Tables

Supplementary Table 1. Photovoltaic performance parameters for PCDTBT:PC70BM solar cells.

PCDTBT fraction (wt%) in PC70BM	*J_{SC} [mA cm^{-2}]*	*V_{OC} [V]*	*FF*	*PCE [%]*
0	***0.2*** ($\pm$0.01)	0.61 ($\pm$0.01)	0.43 ($\pm$0.00)	**0.06** ($\pm$0.00)
0.1	***0.7*** ($\pm$0.04)	0.62 ($\pm$0.04)	0.40 ($\pm$0.03)	**0.2** ($\pm$0.02)
1	***2.9*** ($\pm$0.12)	0.74 ($\pm$0.02)	0.53 ($\pm$0.00)	**1.1** ($\pm$0.06)
5	***9.4*** ($\pm$0.21)	0.76 ($\pm$0.02)	0.44 ($\pm$0.01)	**3.1** ($\pm$0.13)
20	***11.6*** ($\pm$0.2)	0.79 ($\pm$0.02)	0.49 ($\pm$0.02)	**4.5** ($\pm$0.37)
50	***8.7*** ($\pm$0.2)	0.76 ($\pm$0.01)	0.37 ($\pm$0.01)	**2.4** ($\pm$0.08)
80	***1.2*** ($\pm$0.04)	0.93 ($\pm$0.02)	0.23 ($\pm$0.00)	**0.3** ($\pm$0.01)
95	***0.1*** ($\pm$0.00)	0.82 ($\pm$0.03)	0.24 ($\pm$0.00)	**0.03** ($\pm$0.00)
100	***0.04*** ($\pm$0.00)	1.2 ($\pm$0.08)	0.31 ($\pm$0.02)	**0.01** ($\pm$0.00)

The photovoltaic performance parameters including standard errors, such as short-circuit current density (J_{SC}), open circuit voltage (V_{OC}) and fill factor (FF) of the PCDTBT:PC70BM devices with different blend ratios. The presented PCEs correspond to average values of 5-6 pixels on the same device after several JV-measurements (showing stable performance) and also represent the efficiencies of the devices directly before the iPC and IQE measurements were conducted. The error is specified in the brackets and corresponds to the first standard deviation.

Supplementary Table 2. Photovoltaic performance parameters for PTB7:PC70BM solar cells.

PTB7 fraction (wt%) in PC70BM	*J_{SC} [mA cm^{-2}]*	*V_{OC} [V]*	*FF*	*PCE [%]*
10	**2.8** ($\pm$0.07)	0.72 ($\pm$0.01)	0.71 ($\pm$0.01)	**1.4** ($\pm$0.03)
25	**8.1** ($\pm$0.16)	0.75 ($\pm$0.01)	0.60 ($\pm$0.03)	**3.6** ($\pm$0.30)
45	**14.4** ($\pm$0.23)	0.76 ($\pm$0.00)	0.49 ($\pm$0.01)	**5.4** ($\pm$0.10)
65	**5.5** ($\pm$0.73)	0.77 ($\pm$0.02)	0.3 ($\pm$0.02)	**1.3** ($\pm$0.27)
75	***1.9*** ($\pm$0.01)	0.72 ($\pm$0.00)	0.26 ($\pm$0.00)	**0.4** ($\pm$0.01)
82	***1.2*** ($\pm$0.04)	0.71 ($\pm$0.03)	0.26 ($\pm$0.00)	**0.2** ($\pm$0.01)
90	***0.6*** ($\pm$0.01)	0.73 ($\pm$0.03)	0.25 ($\pm$0.01)	**0.1** ($\pm$0.01)
95	***0.4*** ($\pm$0.04)	0.74 ($\pm$0.07)	0.25 ($\pm$0.04)	**0.1** ($\pm$0.01)

The photovoltaic performance parameters including standard errors, such as short-circuit current density (J_{SC}), open circuit voltage (V_{OC}) and fill factor (FF) of the PTB7:PC70BM devices with different blend ratios. The presented PCEs correspond to average values of 3-6 pixels on the same devices after several JV-measurements (showing stable performance) and also represent the efficiencies of the devices directly before the iPC and IQE measurements were conducted. The error is specified in the brackets and corresponds to the first standard deviation.

Supplementary Table 3. Exciton lifetimes, and PLQ as obtained from TAS

PCDTBT fraction (wt%) in PC70BM	$\tau_{exciton}$ **(ps)** [a]	**PLQ** [b]
**0**	600 [4]	-
**1**	1.2	>99
**20**	1.2	>97
**50**	1.2	>97
**80**	1.1	>97
**100**	100	-

(**a**) The exciton decay lifetimes, monitored at the PCDTBT exciton photoinduced absorption of 1300 nm for different PCDTBT:PC70BM blend ratios. (**b**) Exciton quenching of the blend film relative to the corresponding pristine polymer film.

Supplementary References:

[1] Peumans, P., Yakimov, A. & Forrest, S. R. Small molecular weight organic thin-film photodetectors and solar cells. *J. Appl. Phys.* **93,** 3693–3723 (2003).

[2] Zhang, Y. *et al.* Spectral response tuning using an optical spacer in broad-band organic solar cells. *Appl. Phys. Lett.* **102,** 013302 (2013).

[3] Etzold, F. *et al.* Ultrafast exciton dissociation followed by nongeminate charge recombination in PCDTBT:PCBM photovoltaic blends. *J. Am. Chem. Soc.* **133,** 9469–9479 (2011).

[4] Chow, P. C. Y., Albert-Seifried, S., Gélinas, S. & Friend, R. H. Nanosecond intersystem crossing times in fullerene acceptors: Implications for organic photovoltaic diodes. *Adv. Mater.* **26,** 4851–4854 (2014).

[5] Philippa, B. *et al.* The impact of hot charge carrier mobility on photocurrent losses in polymer-based solar cells. *Sci. Rep.* **4,** 5695 (2014).

[6] Stolterfoht, M. *et al.* Photocarrier drift distance in organic solar cells and photodetectors. *Sci. Rep.* **5**, 9949 (2015).

[7] Philippa, B. *et al.* Molecular weight dependent bimolecular recombination in organic solar cells. *J. Chem. Phys.* **141,** 054903 (2014).
