# Supplementary material for: Slower carriers limit charge generation in organic semiconductor light-harvesting systems
Source: Nat Commun. 2016 Jun 21;7:11944. doi: 10.1038/ncomms11944 (PMC4919513; doi:10.1038/ncomms11944)
Supplement: Peer review file [file ncomms11944-s2.pdf]

Editorial Note: this manuscript has been previously reviewed at another journal that is not operating a transparent peer review scheme. This document only contains reviewer comments and rebuttal letters for versions considered at Nature Communications.

Reviewer #1 (Remarks to the Author):

I strongly recommend publication of this manuscript in Nature Communications.
The authors have convincingly responded to all the comments made by the Nature Physics reviewers.
This manuscript will have impact.

Reviewer #2 (Remarks to the Author):

The authors have thoroughly and convincingly addressed my comments, I commend them for that.
I believe the manuscript has gained in clarity and is suitable for publication.
